# Integrated transitional care: patient, informal caregiver and health care provider perspectives on care transitions for older persons with hip fracture

**DOI:** 10.5334/ijic.797

**Published:** 2012-04-13

**Authors:** Justine Toscan, Katie Mairs, Stephanie Hinton, Paul Stolee, The InfoRehab Research Team

**Affiliations:** School of Public Health and Health Systems, University of Waterloo, 200 University Avenue West, Waterloo, ON N2L 3G1, Canada; School of Public Health and Health Systems, University of Waterloo, 200 University Avenue West, Waterloo, ON N2L 3G1, Canada; School of Public Health and Health Systems, University of Waterloo, 200 University Avenue West, Waterloo, ON N2L 3G1, Canada; School of Public Health and Health Systems, University of Waterloo, 200 University Avenue West, Waterloo, ON N2L 3G1, Canada; University of Waterloo, 200 University Avenue West, Waterloo, ON N2L 3G1, Canada

**Keywords:** care transitions, integrated care, musculoskeletal disorders, family-centred care, patient-centred care

## Abstract

**Introduction:**

Complex older adults, such as those with hip fracture, frequently require care from multiple professionals across a variety of settings. Integrated care both between providers and across settings is important to ensure care quality and patient safety. The purpose of this study was to determine the core factors related to poorly integrated care when hip fracture patients transition between care settings.

**Methods:**

A qualitative, focused ethnographic approach was used to guide data collection and analysis. Patients, their informal caregivers and health care providers were interviewed and observed at each care transition. A total of 45 individual interviews were conducted. Interview transcripts and field notes were coded and analysed to uncover emerging themes in the data.

**Results:**

Four factors related to poorly integrated transitional care were identified: confusion with communication about care, unclear roles and responsibilities, diluted personal ownership over care, and role strain due to system constraints.

**Conclusions:**

Our research supports a broader notion of collaborative practice that extends beyond specific care settings and includes an appropriate, informed role for patients and informal caregivers. This research can help guide system-level and setting-specific interventions designed to promote high-quality, patient-centred care during care transitions.

## Introduction

Older adults with chronic illness, deteriorating health status, and dynamic needs frequently require care from multiple professionals across a variety of settings [1, 2]. The term “care transition” describes the movement of a patient between health care providers and settings during the course of a chronic or acute illness [3, 4]. Adair and colleagues define continuity of care as, “a process involving orderly, uninterrupted movement of patients among the diverse elements of the service delivery system” [5, p. 1351]. Unfortunately, care transitions are often fraught with discontinuity and a lack of coordination [3, 6–8], resulting in poor quality care and compromised patient safety [3, 9–12]. Declines in patient health status, re-hospitalization, and increased burden on informal caregivers are common outcomes resulting from poorly executed care transitions [13]. Despite the frequency and importance of transitions for older adults, transitional care has received little attention in research, health policy and clinical practice [3].

Transitional care results in a large number of professionals within and between disciplines and settings, sharing the responsibility of care for one individual, which presents challenges to providing continuous care delivery, particularly for complex older patients [14]. Specifically, it has been observed that as health care teams become more diverse, intergroup relations among members often break down and the perception of team integration decreases [15], resulting in discontinuities in patient care [16]. An integrated model of health care practice has been proposed as a potential solution to fragmented care and has been suggested as critical to improving patient outcomes [17]. Integrated care aims to bring together “services, providers, and organizations from across the continuum to work together jointly so that their services are complementary to one another, are coordinated with each other, and are a seamless unified system, with continuity for the client” [18]. Integrated health care has been linked to a number of potential beneficial outcomes including increased access to services, the quality of care processes and outcomes, patient safety, efficiency of system delivery, and most importantly, the experience of patients and their caregivers [17].

Ineffective communication has been observed to contribute directly to an overall deficiency in providing integrated care [17, 19–22]. Current health literature suggests that a substantial gap exists not only in the communication of patient health information between health care providers both within and across settings [21, 23–25], but also between health care providers and their patients [26, 27]. Patient outcomes have been shown to be significantly influenced by the quality of communication between heath care professionals [28]. Discontinuities in communication can often leave both patients and their informal caregivers unaware, unsure of, or confused about their care plan [19]; unprepared or lacking the information they need to care for themselves or their loved one [19, 27, 29, 30]; and having to contact multiple providers in order to obtain necessary information [26]. Medical complexity is a known predictor of poor transitional care [31]. Older adults with hip fracture, the most common injury requiring the hospitalization of older adults aged 65 years or older [32], often possess multiple co-morbid conditions [33, 34] and represent a particularly complex population. Hip fracture patients often embark on a complicated care trajectory during their rehabilitation, and therefore provide a valuable opportunity to examine the components of transitional care [35–38]. Previous work of our group examined the continuity of information exchange between health care providers across settings relevant to the continuum of care for older hip fracture patients [39]. Our research showed that informational continuity can become unintentionally problematic when an increasingly large number of individuals acting within a patient’s circle of care are responsible for gathering, inputting, sharing and using patient information [39]. We concluded that there is a need to build trusted relationships between care teams in different care settings to promote integrated collaborative practise [39]. Additional work by Suter and colleagues investigated the competencies of collaborative practise considered to be important by frontline health care providers [40]. Two overarching core competencies were identified: understanding and appreciating professional roles and responsibilities; and the need for effective communication. While our previous research along with Suter and colleagues helped to add to an important knowledge deficit surrounding the components of effective interprofessional collaborative practise from the perspective of health care providers [39, 40], it is also important to consider aspects of care integration from multiple perspectives. Consistently including the perspectives of patients and informal caregivers helps ensure that integrated health care delivery systems emphasise a patient-centred, family-focused approach, which meets the needs of all individuals within a patient’s circle of care [5, 41, 42, 43]. Including patients and their informal caregivers in transitional care research is a critical feature of the current study. Methodological and practical challenges such as age-related physiological and psychological declines in both cognition and health [44, 45], particularly for very frail older persons, such as those with hip fracture, are barriers to incorporating their perspectives in research and clinical practise. This is of major concern, as patients and their informal caregivers are often the only common denominator across the care continuum [3] and as a result, often assume major responsibility in the planning, coordination and management of information and care during transitions between settings [46]. In this study, we were able to overcome such barriers, to ensure that patient and caregiver perspectives surrounding integrated care were captured.

### Aims

The objective of the current study was to investigate care coordination for older hip fracture patients from multiple perspectives, including patients, informal caregivers, and health care providers to determine the core factors related to poorly integrated care when patients transition from one care setting to another.

## Methods

### Participants and procedures

The data used for this study were collected at the University of Waterloo as part of a larger pan-national programme of research, InfoRehab Transitions (www.inforehab.uwaterloo.ca). A qualitative, focused ethnographic approach [47] guided this investigation, which is characterised by shorter field visits, large amounts of data, intensive analytic processes, and a greater focus on communicative activities in comparison to classical ethnography [48].

A typical hip fracture patient usually enters the formal health care system through the emergency department, and following a surgical procedure, is admitted to acute care. From this point, the predictability of a patient’s care path often ends, and is largely determined by the formal health care team based on the health characteristics of the patient and early signs of recovery. Next steps and final destinations can include, but are not limited to, in-patient rehabilitation, convalescent care, long-term care and home within the community. In Ontario, a case manager is responsible for coordinating the care of a hip fracture patient as they prepare for discharge from acute care to admission to a subsequent care setting. However, continuity of care is often compromised for the transitioning patient, as the health care teams responsible for discharging the patient from one setting and admitting them into another setting are often different.

Study participants included patients with hip fracture, their informal caregivers and a variety of health care providers. Patients were recruited, based on a specific set of inclusion criteria, which included: diagnosis of hip fracture, over age 65 years, no or very minimal cognitive impairment, and able to read and write English. Patients were first approached to participate in the study by a resource nurse in an acute care hospital setting, who explained the research study and obtained verbal consent from the patient to have a study researcher provide them with further information about the study as a potential participant. During this information session with the researcher, patients were provided with an information letter explaining in greater detail what the study entailed and were given an opportunity to ask questions. Only one individual declined (they felt too overwhelmed as a result of their current health status) following this session (another person passed away following this session, before study participation could begin). No health care providers or family caregivers who were approached to participate declined.

Those who expressed interest in participating following the information session signed a written consent form to be interviewed and observed at each transition point in their care path as they moved across the continuum of care. Based on the patient’s prospective care trajectory, at each point of transition, at least two health care providers involved in the admission or discharge of the patient, along with the patient’s informal caregivers (one or more) were recruited via an information session and written consent process, interviewed and observed.

The interviews for all participants were semi-structured and incorporated both formal, pre-planned questions and flexible probes meant to engage the participants in open-ended discussions about their unique experiences [49]. Separate interview guides were used for patients, health care providers, and family caregivers but explored similar topics including: interactions between formal and informal care team members, information sharing and exchange procedures, processes for communication, and documentation of patient health information (Appendix A). Interviews with patients took place within each care setting they transitioned to, and therefore, several interviews were conducted with each patient participant in the study. Interviews took place at the patient’s convenience, two to seven days following their admission, usually in their room. Health care provider interviews were also conducted in the respective care setting, usually in a staff lounge, office, or cafeteria setting. In order to facilitate a comfortable conversation about a loved one, interviews with the patient’s informal caregivers were conducted at a location of their choice, which ranged from their home to a local coffee shop, to the cafeteria of the respective care setting. Each interview was conducted by one of two trained data collectors, lasted between 20 and 90 minutes, and were recorded and transcribed verbatim [48].

Observations were conducted in 30-minute intervals as many times throughout each patient’s transition process as possible, including: following admission, before and after participant interviews, during discharge, and during time spent in their respective care setting in general, by two trained data collectors. An observation guide and template were used to keep this process consistent across both data collectors. Observations focused on documenting interactions between health care providers, patients and families, and specifically honed in on non-verbal behaviours of the individuals observed, as well as environmental details. Data collectors also recorded their own emotions and reactions to gain an understanding of how their personal views may have affected their interpretation of the data [48, 50].

The study received ethics clearance through the Office of Research Ethics at the University of Waterloo and the Tri-Hospital Research Ethics Board.

### Data analysis

Analyses of interview and observation data took place contemporaneously and followed a hierarchical coding strategy in order to abstract and integrate core concepts from each interview and observation log. Combining the transcript data and field notes provided depth and allowed researchers to analyse the data reflexively using both perspectives [51]. A qualitative data analysis software program, NVivo 8 [52], helped the researchers to organize this process.

Two researchers independently coded the data to enhance the internal validity of the analysis. Using an iterative style, the analysis moved from lower to higher levels of abstraction and identified common patterns and major sub-thematic areas. The multi-step approach began with open coding, whereby researchers highlighted key lines of text in the transcripts related to the integrated team care topic areas targeted in the interview guides. Next, researchers performed axial coding, and categorized the initial open codes according to similarities in the data. Observational notes supplemented this analytic step by providing context for interpreting the interview text. For example, some of the categories that were named included: “communication problems”, “stress”, “boundary issues”, “time constraints”, “lack of responsibility”, “confusion”, and “lack of clarity”. Finally, selective coding aimed to refine and fill out the initial categories in order to generate conclusions that explained what was happening within the data and why [53]. Selective coding involved two independent researchers coming together to reach consensus on the themes emerging from the data. Ongoing iterative meetings were held between the two researchers for several weeks, which were also attended by a third party researcher who provided a non-biased opinion to resolve disagreements. Researchers sought to discover the source and meaning of each category identified, and to assess how consistent these themes were presented across multiple data sources in order to check for analytical completeness and accuracy.

## Findings

In total, six patients who met the inclusion criteria participated, ranging in age from 71 to 94 years, with a mean age of 83 years. Table 1 summarises patient demographics and highlights each transition made by the patient. The number of transitions ranged from one to three (patients were recruited in acute care; therefore, prior care transitions were not included in the data collection). Informal caregivers (n=6) ranged in age from 40 to 70 years. All were female, and most were children of the patient (n=5). In total, 18 health care providers were recruited across four settings. Most were acute care hospital (n=6) and inpatient rehabilitation facility (n=6) employees, while the remainder were from a home care programme (n=4) or retirement home (n=2). Health care providers were predominately case managers (n=6) but also included nurses (n=3), occupational therapists (n=4), physiotherapists (n=4), and a general practitioner (n=1). In total, 45 individual interviews were conducted and over 350 pages of single-spaced transcribed interview data were analysed.

While each group faced unique challenges, collectively, patients, informal caregivers, and health care providers shared in common the experience of poorly integrated transitional care. These shared experiences led to the identification of four core factors:

1. Confusion with communication about care;
2. Unclear roles and responsibilities;
3. Diluted personal ownership over care; and
4. Role strain due to system constraints.

### Confusion with communication about care

Communication breakdown between individuals involved in the circle of care was concerning from all perspectives. For health care providers, incomplete or delayed information transfer was a common concern. One health care provider specifically discussed the occurrence of delayed record keeping procedures when information was being transferred from paper to electronic charts:

“You do have electronic charting… I don’t put it in at the same time that I’m assessing them, so I’ll jot stuff down on paper and then I’ll go in and I’ll document my assessment.”ߞPhysiotherapist

Delays in record keeping may be particularly problematic within the context of transitional care, as transitions are often unexpected, resulting in the need for patient information to be readily available at the time of transition. A delay in record keeping practices could mean that pertinent information may be missing for the patient when needed. As well, the additional process of transferring information from paper to an electronic health record could increase the likelihood of incorrect, inaccurate, or even missed information at the time of electronic input. This could lead to inaccurate or incomplete information being transferred between providers and settings for a transitioning patient. Without current, complete, and up-to-date information available to other professionals and disciplines involved in care, providers were left uncertain and with little trust that procedures or assessments were completed. A registered inpatient rehabilitation nurse described how she always independently conducts a “head to toe” assessment of all newly admitted patients as she is often unaware of what other health professionals have already completed. We further observed health care providers within and across settings showing frustration while waiting for pertinent information to complete their own role in care. For example, during an observation period at a convalescent care home, researchers noted the visible anxiety level of an on-duty nurse, who later explained that her behaviour was a result of the failure of the hospital to forward a list of patient medications in time for their preparation to be complete upon the patient’s arrival. As a result, the nurse was unable to complete a proper admission protocol, having little ability to discuss medication management with the patient.

Informal caregivers were even further removed from the dissemination of information. Uncertainty existed between health care providers and informal caregivers as to who is responsible for initiating communication about care, which led to ambiguity in information sharing and flow. As a result, informal caregivers were often left waiting for information:

“Every time I would ask the nurse she would have to go and look it up to come and give it to me but if I’m talking to somebody I can ask them about medication… how well is he doing in his physiotherapy and they would be able to give me the full information but nobody has time to do that.”—Informal caregiver

Informal caregivers identified proactive strategies as a way to obtain the information they needed. For example, one daughter described becoming so frustrated with the lack of communication between hospital staff and her family that she had to obtain information from a friend who worked within the care setting, but who was not directly involved with her loved one.

Patients also experienced difficulties communicating with members of their care team. For example, patients struggled with physically identifying various health care providers due to a lack of standardised uniforms, and name tags that were not visible. A patient aptly described her issue in obtaining medical information from her care team:

“…it’s not that I don’t remember, I wouldn’t know anyway because you don’t know whether they’re a nurse, health provider or whether they’re just one of the people that serve the meals. You don’t know, because there’s no indication on their uniform.”

### Unclear roles and responsibilities

Patients, informal caregivers, and health care providers demonstrated a lack of clarity in their own individual roles and specific responsibilities, as well as the roles of others within the circle of care. Among health care providers, “blurred boundaries” existed in describing both one’s own professional scope of practise, and that of their colleagues, both within and across care settings. These discrepancies were described as particularly problematic when information was transferred between settings, as it was often unclear who should be responsible for collecting the information, or conversely, when the individual a provider thinks would be responsible for collecting the data, does not in fact collect it. These unclear responsibilities, particularly in a fast-paced clinical environment, could lead to gaps in information exchange within and between settings. As one physician stated:

“The challenge is… the speed of consults and especially discharge summaries getting to me and that’s just a function of the business of the services that are working there right now. Some physicians make it their mandate to make sure they leave without any outstanding charts other physicians not quite so much.”—Physician

As a result of the presence of information gaps between settings, one occupational therapist discussed her solution to call the acute care setting whenever confusion arose:

“I feel very comfortable calling an OT or PT over at the other site and saying you know, asking the questions that might not always be as clear on the chart.”– Occupational therapist

We also observed that a lack of clarity in the responsibilities of care settings involved in patient transfer, as well as between health care providers working within and across settings, greatly influences an informal caregiver’s experience of transitional care. Inconsistencies were often evident during transitions between settings, in the extent of involvement of informal caregivers in care delivery. As they are often the only common individual accompanying patients across settings, we frequently observed a heavy reliance on their assistance, and they were sometimes assumed to take on formal care responsibilities. This often led them to feel stressed and overwhelmed throughout the transition process. For example, one patient’s middle-aged daughter described her frustration over being asked to perform a physical transfer of her mother:

“… so they said something about me making the transfer into the car and I said ‘well I’m not going to do that, I don’t know how to do that, I’m not trained’…I thought it was really a lot that they would expect a family member who’s not trained and is not well to make that kind of a transfer.”—Informal caregiver

Health teams are inconsistent in recognising the most appropriate role of informal caregivers during transitions, which is, according to the informal caregivers interviewed, to provide and receive information, to provide support, and to advocate for their loved one:

“I said you know you really have to let [informal caregiver] and I advocate for you because we’re in a position to do that.”—Informal caregiver

Patients often felt that they were not at the “centre” of their own care. Patients expressed a lack of involvement in the decision-making process regarding their care, and often described feeling as though decisions were made without their input. For example, during her last interview, we asked a patient what advice she would provide to others moving through the formal care system:

“I mean you either are that temperament or not; that you accept or you’re aware that whatever you know, you do what you’re told to do and they say ‘we’re going to do this’ and they do it”—Patient

A case manager further recognised that as a result of a patient not being included or introduced to their care plan and specific responsibilities within a formal care setting, they tend to function poorly upon returning home to the community:

“I know we’ve had people fall through the cracks, kind of waiting to see… what do I do now, I’m home I have all these new medications, I’m starting to have problems with them who do I go to.”—Case manager

### Diluted personal ownership over care

Diluted personal ownership over care was an issue highlighted by health care providers, patients, and their informal caregivers. As patients move through the system, integrated care across settings requires the number of professionals involved in care delivery and management to increase. We observed that as the number of caregivers involved with patient care increases, each individual’s personal ownership or stake in the delivery or outcome of a patient’s care appeared to become diluted.

For health care providers, a greater number of individuals involved in the circle of care may have provided greater opportunity to shift responsibility or to generalise mistakes across the team. For example, a nurse made this comment about her patient being admitted incorrectly using the electronic charting system:

“I think, I don’t know, I just know that the next day they said the admitting messed up and had to fix it and had admitted to the wrong database so it wasn’t our doing.”—Registered nurse

Informal caregivers often felt intimidated or like a burden to the health care team when they required information or clarification on a particular aspect of care. When informal caregivers reflected on their involvement in care, they often wished they had exercised more personal control over the situation by being more assertive with their requests for help and information. For example, during her last interview, when asked what advice she would give to other families, one informal caregiver stated:

“Tell them to be in touch with somebody who was taking care of him all the time. Like don’t wait for them to come and talk to you, just get hold of them and talk to them and talk to them and talk to them because that’s the way to get the information.”—Informal caregiver.

Finally, through discussions with patients, it became clear that they often disengage from the management of their own care. This finding may have emerged as a result of patients perceiving their role as passive in the care transition process, due to the number of individuals involved within their circle of care. Patients expressed feeling overwhelmed, and as a result, lost control and personal autonomy over their care. For example, one patient stated:

“I guess I was just sort of running on [Pause] when you’re thinking of a car sort of running on neutral. I was just sort of running on neutral. I didn’t know what to do. And that was the same at (the acute care hospital); I just didn’t know what was going on. For somebody that has always been in control, I find that very, very difficult.”—Patient

### Role strain due to system constraints

Finally, role strain due to system constraints was a concern from all three perspectives. Throughout the process of discharge planning, health care providers reported feeling pressured to prioritise their patient’s needs, as a result of the policies and procedures specified by the health care system. In one instance, an occupational therapist stated:

**“**I think one of the biggest problems right now that we’re facing is that there is pressure to have people discharged quickly, and there may not always be services available for them when they go home. And a lot of the time we would like to keep people here longer than we do.”—Occupational therapist

Health care providers lacked confidence in discharging their patient to the next care setting due to the expectation of rapid discharge. This had important implications not only for the patient, but also for other key players within a patient’s circle of care.

Informal caregivers often described the unease and stress that they experienced as a result of the increased reliance on informal care placed on them by the system. In one instance, an informal caregiver expressed her concern in providing care to her mother within the home:

“CCAC (Community Care Access Centres) said they would provide that bathing help, they also provide physiotherapy, so a very competent physiotherapist comes in once a week…When the CCAC heard that I was coming to assist Mom they said ‘well then we won’t send anyone for the bathing help’ and that seemed unreasonable given that that was appropriate care for Mom to receive plus I am not as capable or knowledgeable in safe ways to help my Mom.”—Informal caregiver

She later went on to acknowledge the time commitment she and her family allocated to caring for her mother once she transitioned home:

1. Interviewer: “And so you feel like these services that your Mom has are appropriate right now?”
2. Informal caregiver: “Well they’re appropriate given the family member is here for six hours a day at least. Without the family member being here, I don’t believe there’s that kind of constant care available…”

Informal caregivers often did not possess the adequate skills and knowledge to appropriately care for their loved one in the community. One family caregiver reported the stress she endured while caring for her mother while simultaneously dealing with her own medical and personal issues:

“I’d been over three times in six days, you know I’m recovering from major surgery myself and I had a lot of stuff professionally that I was dealing with, so I was pretty whacked out.”—Informal caregiver

These strains may result in the breakdown of collaboration between the individuals involved within the care team, as informal caregivers are left to shoulder major responsibilities for the planning, management and coordination of care across settings.

Finally, patients expressed feeling rushed to recover from their hip fracture and to make important decisions regarding their care. One patient reflected on her first day at a care facility, when she returned to her room to find a sticky note at her bedside with her discharge date from the facility written on it, without recalling any conversations about setting a date. This patient expressed concern about her role in facilitating her own discharge; this anxiety acted as a barrier to her effectively participating in her care network, including communicating her care plan with her family and health care providers.

## Discussion

In recent years, integrated care has emerged as a key priority for health care reform [54, 55], and is particularly relevant for older adults with multiple comorbid conditions [56, 57]. We explored the core factors that contribute to poorly integrated care in the context of transitions between care settings. Four core factors were identified: 1) confusion with communication about care; 2) unclear roles and responsibilities; 3) diluted personal ownership over care; and 4) role strain due to system constraints. Of these factors, two were consistent with those identified by Suter and colleagues, while two novel factors emerged.

Our findings illustrate the influence of poor communication on integrated transitional care. Among health care providers, redundancies and overlap in the collection and sharing of information were most pertinent in impeding effective communication between providers and across settings. Similarly, Suter and colleagues reported that most health professionals agreed that communication needed to be improved within their care setting [40]. Through our study, additional components of communication that facilitate poorly integrated transitional care emerged from the informal caregiver and patient perspectives. Caregivers were uncertain about who should be responsible for initiating communication within and between care settings, and struggled with waiting for important information. Patients were primarily concerned with the identification of different health care providers within their care setting. Overall, our results reinforce communication as an essential aspect of quality integrated, patient-centred care transitional care [6].

Mutually beneficial relationships between health care providers, families and patients are needed to encourage better planning and delivery of services [58]. Our findings illustrate the impact of unclear roles and responsibilities on integrated transitional care. For health care providers, ‘blurred boundaries’ were evident in terms of where the clinical responsibility for a patient ends and begins across care settings. With the decreased likelihood of a single clinician providing continuous care across the continuum [3], it is important that clear boundaries for care are established to maintain continuity. This is similar to the findings of Suter and colleagues, who reported that health care providers acknowledge the importance of understanding individual roles and appreciating the roles of others in promoting effective collaboration, however, many are challenged with the “how to” of such collaboration [40]. They reported that health care providers felt that their role was often misunderstood by other team members, which contributed to tension and resistance surrounding inter-professional collaboration [40].

With the inclusion of patient and informal caregiver perspectives in our research, two factors emerged in addition to those established by Suter and colleagues. The factor, *diluted personal ownership over care*, describes the potential for ownership over care to decrease as the size of the care team increases. The promotion of integrated care teams across settings requires the number of professionals involved in care delivery and management to increase. With the ongoing encouragement of a family-centred approach to care [58], both patient and caregiver perspectives must be considered in care planning. While a collaborative team environment helps ensure a shared vision of health care [59], the dilution of personal ownership may act to impede such integrated practise. Overall, these findings may suggest that a larger circle of care that has not yet achieved an integrated collaborative environment may hinder rather than promote service delivery.

The additional factor of *role strain due to system constraints* recognises the role strain placed on health care providers, patients, and their informal caregivers as a result of system pressures, including the expectation of rapid discharge. Policies supporting rapid discharge can impede integrated transitional care, as patients, informal family caregivers, and health care providers may lack the appropriate time or resources to collaborate on an effective care plan, within and across settings. In the context of limited health care resources and an aging population, frontline care providers with geriatric expertise are in short supply [60]. As a result, informal caregivers are being asked to shoulder the responsibility of planning and coordinating care for their loved ones as they transition through the system. Not only could this over-reliance on informal care result in significant health and safety issues for both caregivers and patients [3, 9–12, 61–63], but it may also lead to further complexity surrounding the appropriate involvement of informal caregivers within the circle of care. Further research is needed to explore the appropriate role for informal caregivers and their potential impact on integrated care for older adults.

The factors identified in this study have the potential to inform the planning and development of both system-level and setting-specific interventions to support an effective collaborative environment. To date, a lack of conceptual clarity surrounding the factors important for integrated transitional care [64] has impeded the development of such interventions. In a recent review of the literature, Gagliardi and colleagues found that while conceptual models for integrated care are well described; health professionals currently work in parallel or consultative models that are not closely integrated, and few interventions have been applied to promote strategies that facilitate further collaboration [65]. Integration throughout the care continuum may be challenged by a number of critical factors, in addition to those uncovered within the current study, including: operational and structural differences between organisations, lack of administrative support and unwillingness to share resources, and power struggles amongst and between teams [65]. As a result, system-level interventions that acknowledge and address such challenges may have the potential to facilitate the shift towards more integrated care [66–68].

The results of this study have the potential to inform several system-level changes that could be implemented to promote integrated transitional care. An important first step may be to recognise that the health care system needs to actively support and encourage integrated care in practise. Specifically, integrated care could be achieved through such strategies as: increased fiscal and human resources available to care partners (e.g. allocating time to collect and share patient information) [43], modifications to job roles and responsibilities to include a specific integrated care team focus (e.g. implementing an organisational structure that includes a working partnership between a physician leader and a non-physician administrator) [69], or the implementation of integrated delivery systems (IDS). The latter intervention strategy has received considerable attention in the recent literature [70]. IDS have been defined as, “a network of organisations that directly provides or arranges to provide a coordinated continuum of services to a defined population, and is able and willing to be held accountable for the cost, quality and outcomes of care, and the health status of the population served” [71, p.7]. Kodner lists the key characteristics of successful IDS programmes, which highlight the need for multidisciplinary or interdisciplinary team care across the entire continuum with a focus on integrated information systems [41]. Shortell and McCurdy further break IDS systems down into two levels of necessary integration: functional integration and clinical integration [72]. Combined, these definitions suggest that integrated health care requires continuity of care through coordinated information systems, cohesive teamwork and a patient-centred focus.

The results of this study could also help to inform several setting-specific changes. Specifically, the introduction of a system navigator role is one recommendation, as their responsibilities help target the requirements for integrated care delivery described by Shortell and colleagues [71], including the coordination and management of care across the continuum, as well as the use of information systems to link patients, health care providers, and informal caregivers [73]. A recent review suggests that older adults transitioning through the health care system would benefit from integrated care directed by a navigator [74]. Navigators could act as a single, constant contact for the patient [73] and help to identify, anticipate, and alleviate barriers experienced by patients during transitional care [74].

Finally, Suter and colleagues identified the role of education in increasing collaborative practise skills [40]. They note the importance of educating students and health care providers about the factors essential for integrated care [40]. Our study results support the need to actively include patients and their informal caregivers in such education as well. Current work within our team has focused on analysing caregiver-specific needs for supporting their loved ones during a transition from hospital to home [75] which may be a starting point for education strategies targeting this population.

Our study has a number of limitations. First, we did not recruit participants with severe cognitive impairment or difficulty communicating in English. These patients are commonly missed in transitional care research and may have unique challenges as they move across the care continuum [10]. Additional investigations are needed to target these groups. Second, although hip fracture patients are a valuable starting point in exploring the integrated transitional care needs of frail, older, medically complex patients, we suggest caution in generalizing our results beyond these patients. Obtaining an adequate sample that is fully representative of the general population is difficult to achieve [76–79], and while our sample yielded a diverse range of patient experiences and care trajectories, it does not reflect all possible patient trajectories. Third, resource nurses who assisted in recruitment reported that some patients who declined participation were dissatisfied and stressed with the health care system, anxious about what participation would entail, or had informal caregivers who were not supportive of participation in research during their recovery. These recruitment barriers have been widely reported [80–82]. Older adults may further be suspicious of research studies due to the fear of negative repercussions or privacy violations, difficulties in understanding instructions, or apprehension in signing forms [82, 83]. Difficulty in recruiting patients was recognised during the data collection phase of this study, and as a result, the recruitment strategy was modified. Lastly, social desirability is a common bias in qualitative research and occurs when participants misrepresent their opinions to make them more consistent with their interpretation of social norms, such as their caregiver’s opinion [84]. Due to environmental factors, such as apartment size and space constraints in hospital rooms, this bias was unavoidable and resulted in informal caregiver presence during some patient interviews. Patients may have modified their opinion to satisfy the caregiver and avoid negative repercussions stemming from their response.

## Conclusions

This study has illustrated the importance of including the perspectives of patients and informal caregivers, as well as health care providers, in understanding the issues and requirements for effective integrated transitional care. Our study reinforces, and also extends, the work of Suter and colleagues to understand the competencies required for effective collaborative practise. It also supports a broader notion of collaborative practise that extends beyond specific care settings and includes an appropriate, informed role for patients and informal caregivers, a crucial priority for addressing the needs of all three perspectives across the continuum of care. We believe that our study results can be helpful in guiding future research, and informing the planning, development and implementation of system-level and setting-specific interventions designed to promote high-quality, patient-centred care.

## Figures and Tables

**Table 1.** Individual patient characteristics

Patient	Gender	Marital status	Residence prior to hip fracture	Transitions
1	Female	Widowed	Home →	Acute care → Inpatient rehab → Retirement home → Home care
2	Male	Widowed	Home →	Acute care → Inpatient rehab → Home care
3	Male	Widowed	Home →	Acute care → Home care
4	Male	Married	Home →	Acute care → Home care
5	Female	Widowed	Home →	Acute care → Inpatient rehab
6	Female	Widowed	Long-term care →	Acute care → Long-term care

## Reviewers

**Aamer Ali**, MRCP, Consultant Physician in Geriatric Medicine, Nottingham University Hospitals NHS Trust, UK

**Ragnhild Hellesø**, RN, PhD, Associate Professor, Faculty of Medicine, Institute of Health and Society, Department of Nursing Sciences, University of Oslo, P.O. Box 1130 Blindern, NO-0318 Oslo, Norway

**Isabelle Vedel**, MD, PhD, Solidage-McGill University-Université de Montreal Research Group on Frailty and Aging, Center for Clinical Epidemiology and Community Studies, Lady Davis Institute for Medical Research, Jewish General Hospital, McGill University, Montreal, Quebec, Canada

## Appendix A: Example interview guide – health care providers

**1. General Background Information**

- Please describe your position here at [INSERT LOCATION, e.g. VGH]?
- How many years have you been employed in this position at this care setting?
- Overall, how many years of experience do you have as a [INSERT POSITION]? (***Probe around specific role during patient transition points, such as admission and discharge; responsibility*)

**2. Patient Transitions – Admission Process **

- Thinking about [PATIENT NAME], please walk me through the steps you are involved in relating to the process of admitting him/her to this unit. I would like to hear about all the people (including health care providers) involved.
  (***Probes: What is your role in this process? Who else is involved? How are they involved? Where are these patients admitted from?*)

**3. Information Exchange – Admission **

- When [INSERT NAME] came to this setting (e.g. unit), what information did you receive from the previous setting (e.g. unit, home, LTC)?
  - How is this information received?
    (***Probe: via email, hard copies, fax, forms, informal communication with health care providers, formal communication or meetings, family care givers, key person etc.*)
- Are there any specific forms that are sent from the previous setting (e.g. unit)?
  - (***IF YES*), can we have a copy of the form(s) received for [SPECIFIC PATIENT]?
- Who is responsible for sending/getting the information to you?
  (***Probe: who gives this information?*)
- Can you tell us about your experiences with the use of electronic records?
- Is there information that you needed about [INSERT PATIENT NAME] from the previous setting that you did not receive?
  - (***IF YES*) Can you give me an example of this?
  - Why do you think that you did not receive this information?
- How do you typically resolve a situation where you do not receive the information needed? (***Probe: did you seek the information you needed, if yes, how and from whom?*)
- What information did you collect from [INSERT PATIENT NAME] once he/she was on this unit?
  (***Probe: past medical history, functional status etc.)*
  - How is this information collected?
    (***Probe for forms, etc.*)
  - Who is this information collected from?
    (***Probe: patient, family*)
- What information did you share with other health care professionals? How is this information shared? *(**Probe: forms, verbal communication etc.*)

**4. Patients/Caregivers Involvement**

- What information did you give to [PATIENT NAME] when he/she arrived on this unit?
  - How was this information provided?
    (***Probe: handouts, around meetings they may have with clinicians, etc.*)
- What information did you give to [PATIENT NAME]'s family/friend care giver when they arrived on this unit?
  - How was this information provided?
    (***Probe: handouts, around meetings they may have with clinicians, etc.*)
- What, if any information is provided by [PATIENT NAME]'s family/friend care givers?
- What, if any, information is provided by [PATIENT NAME]?
- Were there any issues of health literacy and/or language challenges associated with [PATIENT NAME] and/or his/her care giver?
- Were there any advantages when working with [PATIENT NAME]'s family/friend care giver during times of transition.
- Were there any challenges when working with [PATIENT NAME]'s family/friend care giver during times of transition.

5. Discharge Process – Patient Transitions

- Thinking about [PATIENT NAME], please walk me through the steps you are involved in relating to the process of discharging him/her from this unit. I would like to hear about all the people (including health care providers) involved. *(**Probes: What is your role in this process? Who else is involved? How are they involved? Where are these patients discharged to?)*

**6. Information Exchange – Discharge **

- What steps did you take to prepare [PATIENT NAME] for discharge from [CARE SETTING]?
- Did you provide all of the same information to [PATIENT NAME] and his/her care giver?
  - (**IF NO) What information did you give to [PATIENT NAME] before he/she left?
  - (**IF NO) What information did you give to [PATIENT NAME]'s care giver before they left?
- When was this information provided?
- How was this information provided? (***Probe: forms, meetings, etc.*)
- Do you know to what extent are [PATIENT NAME] and his/her care giver were involved in decision making about where he/she was going next?

**7. Concluding questions **

- Thinking about [PATIENT NAME], in your opinion what worked well in terms of their transition to or from your setting (e.g. unit)?
- Are there areas for improvement?
  - (***IF YES*) What?
- What would help you to improve the care of hip fracture patients at times of transition? What do you think can be done to improve how information is sent and received to and from one health care setting to another?
- Is there anything else that you feel is important for us to know about the flow of information for patients who have fractured a hip and those professionals who work with them?
- What would be useful output from our study to help you improve the care of hip fracture patients at times of transition? (***Probe for process maps, steps in the process, improved forms, etc.*)
  - For instance, in other studies, researchers have worked with clinicians, patients, and families to map out the various steps involved during the course of care for a single patient across multiple trajectories. What do you think about this?

**Italics—Notes for interviewer, not to be said to interviewee.
